# A New System for Periprosthetic Fracture Stabilization—A Biomechanical Comparison

**DOI:** 10.3390/jcm11030892

**Published:** 2022-02-08

**Authors:** Daniel Rau, Gabriele Rußow, Mark Heyland, Dag Wulsten, Clemens Kösters, Werner Schmölz, Sven Märdian

**Affiliations:** 1 Center for Musculoskeletal Surgery, Charité-University Medicine Berlin, 13353 Berlin, Germany; gabriele.russow@charite.de; 2 BIH-Julius Wolff Institute for Biomechanics and Musculoskeletal Regeneration, Charité-University Medicine Berlin, 13353 Berlin, Germany; mark.heyland@bih-charite.de (M.H.); dag.wulsten@bih-charite.de (D.W.); 3 Department of Orthopedic, Trauma and Hand Surgery, Maria-Josef-Hospital Greven, 48268 Greven, Germany; Clemens.Koesters@mjh-greven.de; 4 Department of Orthopaedics and Traumatology, Medical University of Innsbruck, 6020 Innsbruck, Austria; Werner.Schmoelz@i-med.ac.at

**Keywords:** periprosthetic femur fracture, biomechanical study, Vancouver B1 fracture, LOQTEQ^®^, locking attachment plate, biomechanical study

## Abstract

In recent years, an increase in periprosthetic femur fractures has become apparent due to the increased number of hip replacements. In the case of Vancouver type B1 fractures, locking plate systems offer safe procedures. This study compared the distal lateral femur plate (LOQTEQ^®^, aap Implantate AG) with a standard L.I.S.S. LCP^®^ (DePuy Synthes) regarding their biomechanical properties in fixation of periprosthetic femur fractures after hip arthroplasty. We hypothesized that the new LOQTEQ system has superior stability and durability in comparison. Eighteen artificial left femurs were randomized in two groups (Group A: LOQTEQ^®^; Group B: L.I.S.S. LCP^®^) and tested until failure. Failure was defined as 10° varus deformity and catastrophic implant failure (loosening, breakage, progressive bending). Axial stiffness, loads of failure, cycles of failure, modes of failure were recorded. The axial stiffness in Group A with 73.4 N/mm (SD +/− 3.0) was significantly higher (*p* = 0.001) than in Group B (40.7 N/mm (SD +/− 2.8)). Group A resists more cycles than Group B until 10° varus deformity. Catastrophic failure mode was plate breakage in Group A and bending in Group B. In conclusion, LOQTEQ^®^ provides higher primary stability and tends to have higher durability.

## 1. Introduction

Many authors agree that endoprosthetic hip replacement is probably the most successful innovation in orthopedic surgery to date [1,2]. With steadily increasing implantation rates of total hip and knee arthroplasty in an equally steadily aging population [3], the incidence of periprosthetic femoral fractures continues to rise [4,5]. Similarly, the increase in life expectancy and a high level of activity, despite advanced age, contribute to the increased incidence of periprosthetic fractures [6]. In older patients, simple trauma usually plays a central role. Due to the different moduli of elasticity of the prosthesis stem and the surrounding bone, stress peaks occur in the area of the stem tip. In this transition area, in particular, these lead to most (approximately 75%) periprosthetic fractures around the hip [7].

Periprosthetic fractures can be classified according to their anatomical localization and divided into intraoperative and postoperative. Known risk factors for intraoperative fracture include severe osteoporosis, osteomalacia, Paget’s disease, rheumatoid arthritis, and cementless implants [8]. Extensive reaming or rasping of the medullary canal may lead to perforation and, thus, to fractures. The incidence of intraoperative fractures is currently reported to be within 0.1–1.0% [9]. Postoperative fractures are observed in 0.15–1.64% of hip replacements. Prosthetic loosening has been identified as a major predisposing factor for periprosthetic fractures [8,10]. These fractures reflect a significant challenge for the surgeon regarding broad surgical experiences and the manufacturers of fracture implants concerning the biomechanical properties of the implants. In particular, the anchorage of osteosynthetic implants at the level of the implanted stem remains a relevant clinical problem.

Cable cerclage systems offer easy handling when fixing a plate in the stem area [11,12]. Biomechanical studies have shown that cable cerclage as an isolated plate fixation method does not guarantee sufficient stability and is inferior to locking screw fixation. However, combining both methods may improve the overall strength [11,12,13,14,15]. Modern polyaxial locking plate systems allow bicortical screw fixation around the prosthetic stem. In a biomechanical study, Gwinner et al. demonstrated that bicortical anchorage is superior to monocortical, but it may lead to a catastrophic failure mode [16]. The authors concluded that preoperative computed tomography is recommended to assess whether bicortical screw anchorage around the stem with the planned screw dimensions is feasible and transcortical anchorage can be avoided. An alternative approach is to combine the plate with attachment plates (e.g., LAP—locking attachment plate, DePuy Synthes, Raynham, MA, USA). Additional locking screws can be inserted to bypass the stem. These techniques led to an expansion of indications for plate fixation in periprosthetic fracture care [17]. However, a major disadvantage of these attachment plates is their monoaxial alignment of the locking screws. The fixed angle for screw placement can lead to monocortical or difficult screw placement and limited stability.

The LOQTEQ^®^ VA Periprosthetic plate system (aap Implantate AG, Berlin, Germany) offers a new concept for the periprosthetic anchorage of locking plates. The plate is extendable by several hinges at the level of the stem. The mobile hinges can be adapted to the patient’s anatomy at angles of up to 45°. Bicortical fixation is easier in the area of the stem due to polyaxially screw fixation with a radius of 30°.

### Purpose

The purpose of this biomechanical study was to test the distal lateral femoral plate of the newly developed LOQTEQ^®^ VA Periprosthetic plate system for stabilizing in Vancouver type B1 fracture versus a standard implant (L.I.S.S. combined with locking attachment plate, Synthes, Raynham, MA, USA). Two hypotheses were tested: (H1) The new LOQTEQ^®^ system will offer increased stability due this a new plate’s morphology and alloy. (H2) The new LOQTEQ^®^ system will be more durable due to differences in the alloy.

## 2. Materials and Methods

### 2.1. Specimen

An in vitro artificial bone model was selected for this study since human cadavers are most often of variable bone density and anatomy and may therefore serve a bias for the results. Eighteen 4th generation left anatomically formed composite femurs (Sawbones, Vashon Island, WA, USA) were used. These have been reported to imitate the biomechanical properties of human bone and are specially developed for biomechanical testing [18,19,20].

### 2.2. Implants

Cementless primary stems (CORAIL^®^, Size 15, DePuy Synthes, Raynham, MA, USA) were implanted following the manufacturer’s guidelines. The hydroxyapatite titanium alloy coating allows a high level of primary stability [21], which is particularly necessary for biomechanical examinations with axial force application. According to their instrumentation, the artificial femurs were randomized into two groups for biomechanical testing. In Group A (n = 9), the 4.5 mm distal lateral femoral plate of the LOQTEQ^®^ VA Periprosthetic plate system was utilized and fixed with two hinges and a total of four locking screws in the stem area. The 4.5/5.0 mm L.I.S.S. LCP^®^ distal femoral plate combined with the 3.5 mm LAP and fixed with four locking screws formed Group B (n = 9).

### 2.3. Surgical Technique

**Figure 1:**
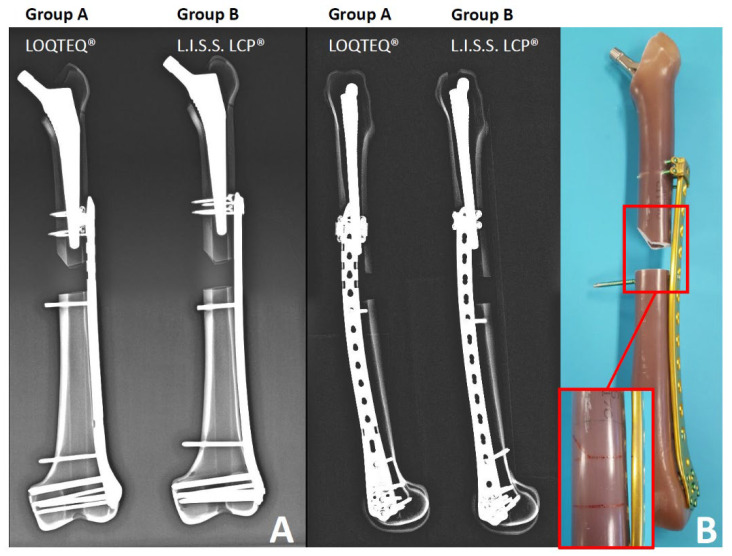
(**A**) Radiological representation of the test construct. (**B**) Osteotomy.

The same surgeon performed all surgical steps. Osteosyntheses were performed after stem implantation but before completing the femoral osteotomy. Thus, the osteosyntheses could be performed identically for all bones. Femoral neck osteotomy was marked on all intact femora (with goniometer applied) to ensure identical osteotomy height and 45° angle. The medullary canal was reamed to size 15 to allow optimal press-fit. Plates of the same length and same screw configuration were used in both groups (Figure 1). In each case, six condylar screws were fixed in the distal fragment. Bicortical locking screws were inserted below the fracture’s distal and proximal holes for maximum stabilization. Therefore the load is concentrated on the proximal anchorage (LAP vs. hinges) using four bicortical 3.5 mm locking screws.

### 2.4. Fracture Model

In an artificial bone model, there is no osteointegration. Therefore osteotomy was adapted accordingly by establishing a Vancouver B1 fracture with a comminution zone below the tip of the stem. This approach is consistent with most established biomechanical studies [7,16,22,23,24,25,26,27,28,29,30]. Following radiological determination of the prosthesis tip and drawing of the osteotomy plane, the femurs were cut orthogonal to the femoral shaft axis. A segment of 10 mm length was removed. The medial cortex was angled 45° to prevent medial support during testing.

### 2.5. Biomechanical Testing

**Figure 2:**
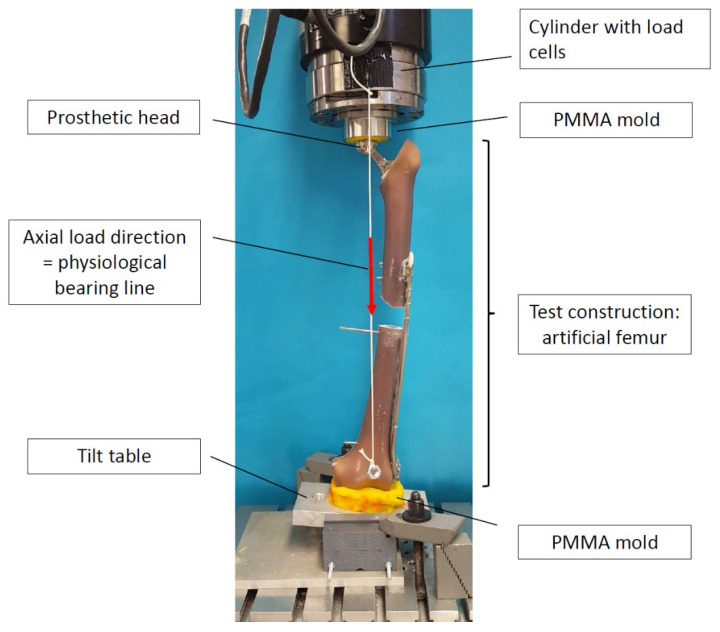
Test setup.

An experimental setup similar to that described in previous studies [16,30,31] was used. However to allow a more physiological and less constraint loading the fixation of the specimen in the test machine was modified. Unlike the aforementioned studies, the present study utilized a pendulum to determine the weight-bearing line and placed specimens freely. Therefore a prosthetic head (CoCr Ø 28/0 M) was placed on the stem. The specimen was mounted in a mobile manner under the load cell. Custom-made polymethyl methacrylate molds (PMMA, Technovit 4000, Heraeus, Hanau, Germany) and an additional tilting table simulated the physiological movement in the knee joint (Figure 2).

Analyses of load and moment transmission showed that the femoral cortex is mainly loaded axially along the load-bearing line during walking [32]. Therefore, the axial load was chosen as the main parameter. A pendulum was applied to determine the physiological load-bearing line. Testing was performed with a servo-hydraulic testing machine (MTS 852 Mini Bionix II, Eden Prairie, MN, USA) and recorded using the supplied test apparatus software (TestStar, MTS).

Preconditioning with constant axial preload of 100 N was applied for 10 s before each testing. Followed by sinusoidal axial loading starting with an initial peak load of 600 N and successively increasing the peak load by 50 N every 500 load cycles until failure. The lower load during sinusoidal loading was kept constant (100 N). This approach was chosen to simulate repetitive loading during normal walking (loading frequency 1 Hz) with stepwise increasing weight-bearing during a patient’s clinical course.

### 2.6. Data Collection

Time, number of cycles, axial failure load, and axial deformation of the test specimen were recorded at a frequency of 50 Hz using the test software (TestStar, MTS, Eden Prairie, MN, USA). In addition, the failure pattern was photo-documented and recorded.

Axial stiffness was calculated by fitting a linear function into each individual loading and unloading curve (cycle) using all data points in this part of the curve (loading or unloading) with a macro in Microsoft^®^ Excel^®^, Version 2016 (Microsoft, Richmond, VA, USA). Then mean slope of those curves for 500 cycles was calculated as axial stiffness. Based on prior studies, statistical power was estimated a priori a number of 9 specimens each group with an effect size of 1.7 [33,34]. The test power was set at (1 − β) ≥ 0.8 and the significance level at α = 0.05 or 0.025 for the primary endpoints adapted by the Bonferroni method. Results were examined to normal distribution performing the Kolmogorov–Smirnov test and graphical analysis. Unpaired Student *t*-tests and, the Mann–Whitney U tests were conducted to test primary outcomes. Data were analyzed using Microsoft^®^ Excel^®^, version 2016 (Microsoft, Richmond, VA, USA), IBM^®^ SPSS^®^ Statistic Release 27.0 (IBM Corporation, Armonk, NY, USA) and G*Power Version 3.1.9.4 (Christian-Albrechts-Universität, Kiel, Germany).

### 2.7. Modes of Failure

Modes of failure were defined as follows:

(1) Deformation of more than 10° varus deformity after completion of a loading cycle [28,30].
(2) Breakage of the implant.
(3) Loosening of the implant.

## 3. Results

A total of seventeen samples completed testing. Of note, we excluded one sample because the saw bone construct showed an early fatal failure at cycle 393. The cause was a mistake during the osteosynthesis in the LAP area and was not included in analyses.

### 3.1. Axial Stiffness

**Figure 3:**
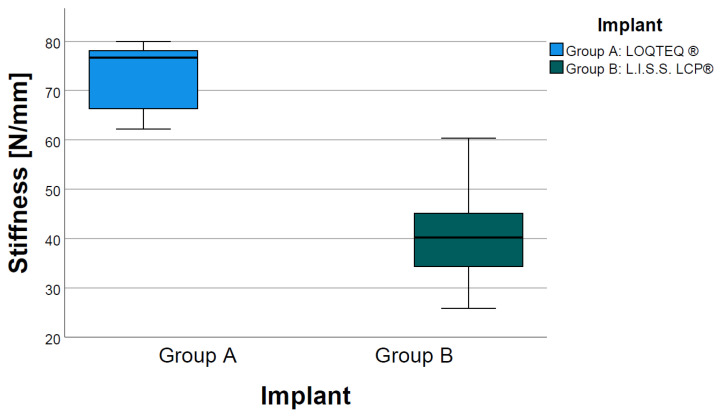
Statistical comparison of axial stiffness.

Mean axial stiffness was significantly higher (*p* = 0.001) in Group A (LOQTEQ^®^) with 73.4 N/mm (SD +/− 6.9) compared to Group B (L.I.S.S. LCP^®^) with 40.7 N/mm (SD +/− 9.8) (Figure 3).

### 3.2. Modes of Failure

Taking a deformation of 10° as failure, Group A withstood with a mean failure cycle of 2203.2 (SD +/− 208.4; range 1857 to 2427 cycles), more cycles than Group B with 1763.6 (SD +/− 564.8, range 1000 to 2500 cycles). However, there was no significant difference in the failure cycle (*p* = 0.209), although the range of cycles in Group A was substantially smaller than in Group B. Thus, failure was more predictable based on this defined criterion. The recorded failure load at 10° plastic deformation was quantitively higher in Group A with 752.9 N (SD +/− 21.1) than in Group B with 689.7 N (SD +/− 63, 7); however, a significant difference was not present (*p* = 0.093). No specimen in either group showed a catastrophic failure (breakage or loosening) before plastic deformation of 10°.

Catastrophic failure occurred at a mean failure cycle of 2773.3 (SD +/− 167.9) and a maximum load of 781.0 N (SD +/− 19.1) in Group A and at 2706.4 (SD +/− 323.1) and 747.9 N (SD +/− 81.1) in Group B. A significant difference between failure cycle (*p* = 0.630) and failure load (*p* = 0.178) was not observed. Only the failure pattern in the catastrophic failure test differed significantly in the two groups. In Group A, the implant broke in 7 of 9 cases (78%). In all specimens of Group B, the plate showed a bending. No screw loosening was observed in both groups.

## 4. Discussion

The most crucial goal in the treatment of periprosthetic fractures is rapid, pain-free mobilization of the patient, because analogous to the "classic" proximal femoral fractures, the mortality risk from suffering such a fracture is high at up to 27% [35,36,37,38]. High primary stability and sufficient fracture healing are fundamental [7,39,40,41]. In our biomechanical study, we were able to show that the LOQTEQ^®^ hinge system had a significantly higher axial stiffness than the control group of the standard implant. A direct comparison of the absolute values of the axial stiffness with other biomechanical studies is difficult due to the different setups and calculation methods. However, there were no outliers among the respective study groups A and B. These results align with the context of previous similar studies [7,26,28,29,30,42], which supports the Hypothesis (H1) that the LOQTEQ^®^ offers higher stability. In direct comparison to the study by Wähnert et al. 2020 [29], the stiffness for the LOQTEQ^®^ was approximately 3.5 times higher than in our study (253.9 N/mm (SD +/− 46.8) vs. 73.4 N/mm (SD +/− 3.0)). The higher stiffness is explained by a significantly longer fixation distance and two additional monocortical, 5.0 mm locking screws in the stem area. The free working length is one of the most important factors influencing the resulting mechanobiology of a locking plate osteosynthesis. A longer working length leads to a reduced stiffness of the construct and larger interfragmentary movements in the fracture gap [43,44]. Furthermore, the comparative study determined the axial stiffness over ten cycles (cycles 10 to 19) [28,29,30]. It probably led to the significant discrepancy in stiffness for the LCP^®^ in his studies from 2014 (47 N/mm +/− 12) [30] and 2020 (145.6 N/mm +/− 27.1) [29] under similar test conditions. During the data recording, we saw that the stiffness was relatively high or even increasing in the early cycles but then continuously settled in the course of the cyclical testing. Therefore the first 500 cycles were used to determine axial stiffness in our experiment.

Wähnert et al. [29,30] determined the axial stiffness using the minimum and maximum of a cycle in the force-displacement diagram. In our study, the mean slope of the curves for 500 cycles—as described above—was calculated as axial stiffness, which, in our opinion, leads to more precise results. [7]. Nevertheless, both studies report that the LOQTEQ^®^ plate has a significantly higher stiffness compared to the control group (LCP^®^).

Similar to previous studies, the failure mode was defined as a varus of 10° [29,31] as well as a plate breakage or a progressive bending (catastrophic failure) [7,26,29,42]. Our study shows that Group A withstood about 25% more cycles than Group B until 10° of plastic deformation. It also corresponds to the observations made in the biomechanical study by Wähnert et al. [29]. The corresponding failure load of 752.9 N +/− 21 is slightly advantageous for Group A over the standard implant with 689.7 N +/− 63.7. While we were not able to demonstrate significance in terms of durability as per Hypothesis (H2), increasing the sample size may potentially achieve this. Nevertheless, the 25% longer durability in group A could influence the implant choice in favor of the LOQTEQ^®^ system. A delayed union may—of course, depending on other physiological factors—be observed for longer and then lead eventually to a fracture healing without having to fear an early implant failure. However, both the number of cycles and the failure load showed that the results for Group A exhibit a significantly lower scatter. Therefore, a stiffer metal alloy may most likely explain the higher failure load. The assumption that the alloy plays a role in terms of stability (H1) is also supported because, despite the identical osteosynthesis configuration in both groups, the stiffness results in Group A are significantly higher. It also means that the failure of the osteosynthesis in Group A appears more validly predictable due to the minor variation.

The test for catastrophic failure showed no differences in failure cycle and failure load in the two groups, but the test revealed a substantial difference in the failure mode. Nearly all implants in Group A failed in breakage (n = 7 out of 9), whereas none of the implants in Group B broke. In their study, Wähnert et al. [29,30] observed the same failure pattern for the LCP^®^ with the bending of the plate, whereas the LOQTEQ^®^ initially withstood the stress for a more extended period and then eventually broke. Possible causes could be differences in the alloy or the plate morphology. The NCB^®^ PP (Non-Contact Bridging Periprosthetic Proximal Femur Plate, Zimmer Biomet, Warsaw, IN, USA) offers another concept. Due to additional plate-dependent options, cortical and polyaxial locking screw fixation is enabled. The direct comparison with the L.I.S.S. by Konstantinidis et al. [42] showed no advantage of either of these plate systems. However, the number of samples with five pairs of cadaver specimens was chosen too small. In contrast, the NCB^®^ showed significantly superior axial stiffness in the cadaver study by Wähnert et al. [30], similar to the LOQTEQ^®^ plate system.

Besides, other systems for periprosthetic fracture management do exist on the market. For example, the non-contact bridging plate system (NCB^®^) offers the possibility of polyaxial screw placement through the primary implant. However, the LOQTEQ^®^ plate may provide more options regarding the achievable angulation that may be realized around the stem. Due to the adjustable hinges, a wider range of screw placement may be realized. For our study, we chose the L.I.S.S. plus attachment plate for two reasons: First, the L.I.S.S. is still the gold standard. So, new systems have to benchmark against it. Second, the screw diameter for the hinges and the main implants are identical, making a direct comparison feasible.

This present study utilized a previously published test setup [16,30,31]. Modes of failure observed in this study, including bending [23,45], and torsional forces [13] have been previously documented. Duda et al. [32] carried out analyses of the force and torque transmission and determined that during normal walking, the femoral cortex is stressed primarily axially along the supporting line. The bending and torsional forces were relatively negligible. Our study incorporated these considerations into our test setup, which was guided by previous studies [16,28,29,30]. The positioning of test samples, however, varied across studies. Most authors utilized a varus position in the range of mostly 6° to 15° depending on the test setup [7,26,28,29,30,46]. In comparison with other studies, the present study determined the load-bearing line by using a pendulum and placing specimens freely in pre-made molds. This technique may reduce undesired shear forces due to constraints in the specimen fixation and therefore reduced a possible error in the calculated construct stiffness. However, in the case of the embedded specimen, an additional articulation in the “hip or knee joint” is not considered [7,23,26].

Two main approaches have emerged regarding the principle of load transmission in the last few years. One relies on quasi-static loading [7,13,24,25,27,45,47,48,49], whereas the other uses cyclic loading until failure [14,15,23,26,30,42,50]. The cyclical testing represents the stress and relief during normal walking. The step-by-step increase in the preload serves for a better physiological test of an initial partial weight-bearing with continuous load adding in the postoperative phase [33]. From clinical experience, the implant failure is already signaled by bending. Therefore a permanent 10° varus deformity was defined as an implant failure. The failure mode was determined by continuing the cyclical loading [29,30,31].

The Vancouver type B1 fracture, being the most common periprosthetic fracture and the most frequently used fracture type in biomechanical testing of periprosthetic femoral fractures, was used for better comparability for our study [7,13,14,15,23,24,25,30,33,39,42,45,47,48,49,50,51,52,53,54]. From clinical experience, the stem in this fracture type is still firmly embedded in the femoral shaft and does not need to be changed. Thus, the Vancouver type B1 fractures do not occur due to loosening but rather due to adequate trauma (e.g., fall or traffic accident) [8] and are usually surgically stabilized in the clinical care algorithm using locking plate fixation.

An incorrect decision due to poor fracture analysis carries a high risk of early failure of the osteosynthesis and further perioperative complications. It poses immense challenges for the surgeon in managing such injuries [7]. The fixation of the plate systems in the stem area is still one of the main challenges of periprosthetic fracture treatment. Various possible combinations with attachment plates and cerclages have been developed for the standard implants. Many biomechanical studies have summarized that bicortical screw fixation in the area of the prosthesis bed is superior to monocortical fixation [16,22] and the use of cable cerclages [11,13,15,49,55].

The distal lateral femoral plate of the LOQTEQ^®^ plate system offers an innovative implant for the operative stabilization of periprosthetic fractures of the femur. It may be implanted in a minimally invasive manner, thus complying with the concept of biological osteosynthesis. Furthermore, the modular nature of the hinges allows fixing them at different angles (up to 45°). Additional options of multidirectional insertion of locking screws offer the surgeon optimal options for securely anchoring the plate in the stem area [29].

In summary, direct comparison across biomechanical studies is difficult due to the heterogeneity in approaches investigated [22,33].

Despite the strengths of this study, including the ability to directly compare modes of failure via simulated weight-bearing, the following limitations were determined:

(1) Only synthetic femurs were used. Older adults [56,57] experience periprosthetic femur fractures due to prosthetic loosening, trauma, and less bone quality at higher rates than younger adults [4,5]. We know that screw anchoring in the bone depends mainly on the quality of the bone [34,48,58,59,60]. Using synthetic femurs, however, provided the present study with improved reproducibility, decreased cost (e.g., a smaller number of samples necessary), improved availability and acquisition, and improved efficiency in the study design as cold storage and preparation were not required.
(2) Another limitation is the small sample size of the present study. Ideally, a higher number of specimens and, therefore, a higher power level, would be desirable; however, this would have significantly increased study cost. Nevertheless, a smaller number of samples was utilized and deemed acceptable due to a minor standard deviation reported by previous studies [7,26,28,33,34].
(3) Only the axial load was tested in the present study. However, other loads also act on the osteosynthesis during everyday activities, such as torsional or shear forces. The study does not consider influencing factors, such as muscle tension and soft tissue. Overall, the axial testing in biomechanical studies remains sufficient because studies suggest that the axial load is the main component of loading forces, while other forces are neglectable [32,61].
(4) The present biomechanical study represented an abstract simulation of reality, and the results need to be interpreted in the context of clinical experience. Additionally, reviews highlight that use of heterogeneous testing techniques which make a direct comparison across studies difficult [22].
(5) The present study only investigated two types of implant failure. In clinical practice, complete fracture or loosening of the plate does not always occur. Relevant permanent deformations of the implant are also equivalent to a failure.

The degree of varus deformity taken as endpoint for construct failure varies in the literature. While some studies consider 10° as failure, a recent study already considered 5° of varus deformity as failure [29]. Currently, there is no unanimous opinion as to when this endpoint has been reached.

## 5. Conclusions

Compared to the previous standard, the innovative hinge design proved that it is at least equal to the standard in terms of failure load and time to failure. Our experiment also reported the superiority of the new implant in terms of primary stability. The current study underscores the ongoing complexity of treating periprosthetic femoral fractures.

## Data Availability

The data that support the findings of this study are available from D.R. and S.M. Restrictions apply to the availability of these data, which were used under license for this study.

## References

[1] Callaghan J.J., Albright J.C., Goetz D.D., Olejniczak J.P., Johnston R.C. (2000). Charnley Total Hip Arthroplasty with Cement: Minimum twenty-five-year follow-up. J. Bone Jt. Surg..

[2] Learmonth I.D., Young C., Rorabeck C. (2007). The operation of the century: Total hip replacement. Lancet.

[3] Wengler A., Nimptsch U., Mansky T. (2014). Hip and knee replacement in Germany and the USA: Analysis of individual inpatient data from German and US hospitals for the years 2005 to 2011. Dtsch. Arztebl. Int..

[4] Stoffel K., Sommer C., Kalampoki V., Blumenthal A., Joeris A. (2016). The influence of the operation technique and implant used in the treatment of periprosthetic hip and interprosthetic femur fractures: A systematic literature review of 1571 cases. Arch. Orthop. Trauma. Surg..

[5] Meek R.M.D., Norwood T., Smith R., Brenkel I.J., Howie C.R. (2011). The risk of peri-prosthetic fracture after primary and revision total hip and knee replacement. J. Bone Jt. Surgery. Br. Vol..

[6] Thien T.M., Chatziagorou G., Garellick G., Furnes O., Havelin L.I., Mäkelä K., Overgaard S., Pedersen A., Eskelinen A., Pulkkinen P. (2014). Periprosthetic femoral fracture within two years after total hip replacement: Analysis of 437,629 operations in the nordic arthroplasty register association database. J. Bone Jt. Surg. Am..

[7] Hoffmann M.F., Burgers T.A., Mason J.J., Williams B., Sietsema D.L., Jones C.B. (2014). Biomechanical evaluation of fracture fixation constructs using a variable-angle locked periprosthetic femur plate system. Injury.

[8] Gruner A., Hockertz T., Reilmann H. (2004). Periprosthetic fractures: Classification, management, therapy. Unfallchirurg.

[9] Kavanagh B.F. (1992). Femoral fractures associated with total hip arthroplasty. Orthop. Clin. N. Am..

[10] Wick M., Muller E.J., Kutscha-Lissberg F., Hopf F., Muhr G. (2004). Periprosthetic supracondylar femoral fractures: LISS or retrograde intramedullary nailing? Problems with the use of minimally invasive technique. Unfallchirurg.

[11] Wähnert D., Lenz M., Schlegel U., Perren S., Windolf M. (2011). Cerclage handling for improved fracture treatment. A biomechanical study on the twisting procedure. Acta Chir. Orthop. Traumatol. Cechoslov..

[12] Raschke M., Stange R., Kösters C. (2012). Versorgung periprothetischer und periimplantärer Frakturen. Unfallchirurg.

[13] Lenz M., Perren S.M., Gueorguiev B., Höntzsch D., Windolf M. (2013). Mechanical behavior of fixation components for periprosthetic fracture surgery. Clin. Biomech..

[14] Lenz M., Perren S.M., Gueorguiev B., Richards R.G., Hofmann G.O., Dell’Oca A.F., Höntzsch D., Windolf M. (2014). A biomechanical study on proximal plate fixation techniques in periprosthetic femur fractures. Injury.

[15] Lenz M., Perren S.M., Richards R.G., Mückley T., Hofmann G.O., Gueorguiev B., Windolf M. (2012). Biomechanical performance of different cable and wire cerclage configurations. Int. Orthop..

[16] Gwinner C., Märdian S., Dröge T., Schulze M., Raschke M.J., Stange R. (2015). Bicortical screw fixation provides superior biomechanical stability but devastating failure modes in periprosthetic femur fracture care using locking plates. Int. Orthop..

[17] Wähnert D., Schliemann B., Raschke M., Kösters C. (2014). Versorgung periprothetischer Frakturen. Orthopäde.

[18] Chong A.C.M., Miller F., Buxton M., Friis E.A. (2007). Fracture Toughness and Fatigue Crack Propagation Rate of Short Fiber Reinforced Epoxy Composites for Analogue Cortical Bone. J. Biomech. Eng..

[19] Chong A.C.M., Friis E.A., Ballard G.P., Czuwala P.J., Cooke F.W. (2007). Fatigue Performance of Composite Analogue Femur Constructs under High Activity Loading. Ann. Biomed. Eng..

[20] Zdero R., Olsen M., Bougherara H., Schemitsch E.H. (2008). Cancellous bone screw purchase: A comparison of synthetic femurs, human femurs, and finite element analysis. Proc. Inst. Mech. Eng. Part H J. Eng. Med..

[21] Vidalain J.-P. (2010). Twenty-year results of the cementless Corail stem. Int. Orthop..

[22] Wang K., Kenanidis E., Miodownik M., Tsiridis E., Moazen M. (2018). Periprosthetic fracture fixation of the femur following total hip arthroplasty: A review of biomechanical testing—Part II. Clin. Biomech..

[23] Choi J.K., Gardner T.R., Yoon E., Morrison T.A., Macaulay W.B., Geller J.A. (2010). The effect of fixation technique on the stiffness of comminuted Vancouver B1 periprosthetic femur fractures. J. Arthroplast..

[24] Dennis M.G., Simon J.A., Kummer F.J., Koval K.J., DiCesare P.E. (2000). Fixation of periprosthetic femoral shaft fractures occurring at the tip of the stem: A biomechanical study of 5 techniques. J. Arthroplast..

[25] Moazen M., Leonidou A., Pagkalos J., Marghoub A., Fagan M.J., Tsiridis E. (2016). Application of Far Cortical Locking Technology in Periprosthetic Femoral Fracture Fixation: A Biomechanical Study. J. Arthroplast..

[26] Sariyilmaz K., Dikici F., Dikmen G., Bozdag E., Sunbuloglu E., Bekler B., Yazicioglu O. (2014). The Effect of Strut Allograft and Its Position on Vancouver Type B1 Periprosthetic Femoral Fractures: A Biomechanical Study. J. Arthroplast..

[27] Sarıyılmaz K., Korkmaz M., Özkunt O., Gemalmaz H.C., Sungur M., Baydoğan M. (2015). Comparison of fixation techniques in Vancouver type AG periprosthetic femoral fracture: A biomechanical study. Acta Orthop. Traumatol. Turc..

[28] Wähnert D., Grüneweller N., Gehweiler D., Brunn B., Raschke M.J., Stange R. (2016). Double plating in Vancouver type B1 periprosthetic proximal femur fractures: A biomechanical study. J. Orthop. Res..

[29] Wähnert D., Müller M., Tiedemann H., Märdian S., Raschke M.J., Kösters C. (2020). Periprosthetic fracture fixation in Vancouver B1 femoral shaft fractures: A biomechanical study comparing two plate systems. J. Orthop. Transl..

[30] Wähnert D., Schröder R., Schulze M., Westerhoff P., Raschke M., Stange R. (2013). Biomechanical comparison of two angular stable plate constructions for periprosthetic femur fracture fixation. Int. Orthop..

[31] Märdian S., Schmölz W., Schaser K.-D., Duda G.N., Heyland M. (2015). Interfragmentary lag screw fixation in locking plate constructs increases stiffness in simple fracture patterns. Clin. Biomech..

[32] Duda G.N., Schneider E., Chao E.Y. (1997). Internal forces and moments in the femur during walking. J. Biomech..

[33] Moazen M., Jones A.C., Jin Z., Wilcox R.K., Tsiridis E. (2011). Periprosthetic fracture fixation of the femur following total hip arthroplasty: A review of biomechanical testing. Clin. Biomech..

[34] Cristofolini L., Viceconti M., Cappello A., Toni A. (1996). Mechanical validation of whole bone composite femur models. J. Biomech..

[35] Bhattacharyya T., Chang D., Meigs J.B., Estok D.M., Malchau H. (2007). Mortality after periprosthetic fracture of the femur. J. Bone Jt. Surg. Am..

[36] Drew J.M., Griffin W.L., Odum S.M., Van Doren B., Weston B.T., Stryker L.S. (2015). Survivorship after Periprosthetic Femur Fracture: Factors Affecting Outcome. J. Arthroplast..

[37] Matharu G.S., Pynsent P.B., Dunlop D.J., Revell M.P. (2012). Clinical Outcome following Surgical Intervention for Periprosthetic Hip Fractures at a Tertiary Referral Centre. HIP Int..

[38] Streubel P. (2013). Mortality after Periprosthetic Femur Fractures. J. Knee Surg..

[39] Rupprecht M., Sellenschloh K., Grossterlinden L., Püschel K., Morlock M., Amling M., Rueger J.M., Lehmann W. (2011). Biomechanical Evaluation for Mechanisms of Periprosthetic Femoral Fractures. J. Trauma Inj. Infect. Crit. Care.

[40] Tan S.E., Balogh Z.J. (2009). Indications and limitations of locked plating. Injury.

[41] Ricci W.M. (2015). Periprosthetic femur fractures. J. Orthop. Trauma.

[42] Konstantinidis L., Hauschild O., Beckmann N., Hirschmüller A., Südkamp N., Helwig P. (2010). Treatment of periprosthetic femoral fractures with two different minimal invasive angle-stable plates: Biomechanical comparison studies on cadaveric bones. Injury.

[43] Märdian S., Schaser K.-D., Duda G.N., Heyland M. (2015). Working length of locking plates determines interfragmentary movement in distal femur fractures under physiological loading. Clin. Biomech..

[44] Stoffel K., Dieter U., Stachowiak G., Gächter A., Kuster M.S. (2003). Biomechanical testing of the LCP—How can stability in locked internal fixators be controlled?. Injury.

[45] Lever J.P., Zdero R., Nousiainen M.T., Waddell J.P., Schemitsch E.H. (2010). The biomechanical analysis of three plating fixation systems for periprosthetic femoral fracture near the tip of a total hip arthroplasty. J. Orthop. Surg. Res..

[46] Gordon K., Winkler M., Hofstädter T., Dorn U., Augat P. (2016). Managing Vancouver B1 fractures by cerclage system compared to locking plate fixation—A biomechanical study. Injury.

[47] Siddiqui F.S., Shah S., Nicayenzi B., Schemitsch E.H., Zdero R., Bougherara H. (2013). Biomechanical analysis using infrared thermography of a traditional metal plate versus a carbon fibre/epoxy plate for Vancouver B1 femur fractures. Proc. Inst. Mech. Eng. Part H J. Eng. Med..

[48] Demos H.A., Briones M.S., White P.H., Hogan K.A., Barfield W.R. (2012). A Biomechanical Comparison of Periprosthetic Femoral Fracture Fixation in Normal and Osteoporotic Cadaveric Bone. J. Arthroplast..

[49] Dennis M.G., Simon J.A., Kummer F.J., Koval K.J., Di Cesare P.E. (2001). Fixation of Periprosthetic Femoral Shaft Fractures: A Biomechanical Comparison of Two Techniques. J. Orthop. Trauma.

[50] Pletka J.D., Marsland D., Belkoff S.M., Mears S.C., Kates S.L. (2011). Biomechanical Comparison of 2 Different Locking Plate Fixation Methods in Vancouver B1 Periprosthetic Femur Fractures. Geriatr. Orthop. Surg. Rehabil..

[51] Graham S.M., Mak J.H., Moazen M., Leonidou A., Jones A.C., Wilcox R., Tsiridis E. (2015). Periprosthetic femoral fracture fixation: A biomechanical comparison between proximal locking screws and cables. J. Orthop. Sci..

[52] Zdero R., Walker R., Waddell J.P., Schemitsch E.H. (2008). Biomechanical Evaluation of Periprosthetic Femoral Fracture Fixation. J. Bone Jt. Surg..

[53] Fulkerson E., Koval K., Preston C.F., Iesaka K., Kummer F.J., Egol K.A. (2006). Fixation of periprosthetic femoral shaft fractures associated with cemented femoral stems: A biomechanical comparison of locked plating and conventional cable plates. J. Orthop. Trauma.

[54] Wilson D., Frei H., Masri B.A., Oxland T.R., Duncan C.P. (2005). A biomechanical study comparing cortical onlay allograft struts and plates in the treatment of periprosthetic femoral fractures. Clin. Biomech..

[55] Lenz M., Windolf M., Mückley T., Hofmann G.O., Wagner M., Richards R.G., Schwieger K., Gueorguiev B. (2012). The locking attachment plate for proximal fixation of periprosthetic femur fractures—A biomechanical comparison of two techniques. Int. Orthop..

[56] Moreta J., Aguirre U., de Ugarte O.S., Jáuregui I., Mozos J.L.M.-D.L. (2015). Functional and radiological outcome of periprosthetic femoral fractures after hip arthroplasty. Injury.

[57] Masri B.A., Meek R.M.D., Duncan C.P. (2004). Periprosthetic Fractures Evaluation and Treatment. Clin. Orthop. Relat. Res..

[58] Wähnert D., Hoffmeier K.L., Klos K., Stolarczyk Y., Fröber R., Hofmann G.O., Mückley T. (2010). Biomechanical Characterization of an Osteoporotic Artificial Bone Model for the Distal Femur. J. Biomater. Appl..

[59] Heiner A.D., Brown T.D. (2001). Structural properties of a new design of composite replicate femurs and tibias. J. Biomech..

[60] Papini M., Zdero R., Schemitsch E.H., Zalzal P. (2006). The Biomechanics of Human Femurs in Axial and Torsional Loading: Comparison of Finite Element Analysis, Human Cadaveric Femurs, and Synthetic Femurs. J. Biomech. Eng..

[61] Taylor M.E., Tanner K.E., Freeman M.A., Yettram A.L. (1996). Stress and strain distribution within the intact femur: Compression or bending?. Med. Eng. Phys..

